# FORECASTING PREVALENCE AND MORTALITY OF ALZHEIMER’S DISEASE AND RELATED DEMENTIAS USING PARTITIONING MODELS

**DOI:** 10.1093/geroni/igac059.1830

**Published:** 2022-12-20

**Authors:** Igor Akushevich, Arseniy Yashkin, Mikhail Kovtun, Julia Kravchenko, Konstantin Arbeev, Anatoliy Yashin

**Affiliations:** Duke University, Durham, North Carolina, United States; Duke University, Durham, North Carolina, United States; Duke university, Durham, North Carolina, United States; Duke University School of Medicine, Durham, North Carolina, United States; Duke University, Durham, North Carolina, United States; Duke University, Durham, North Carolina, United States

## Abstract

A forecasting model for prevalence and mortality based on a trend partitioning approach which models trends in age-adjusted prevalence and incidence-based mortality in terms of changes in interpretable epidemiologic quantities such as disease incidence and survival, is developed and applied to generate forecasts of Alzheimer’s disease (AD) and related dementia (ADRD) prevalence and mortality up to 2035 using health data drawn from a 5% sample of the total Medicare population. Forecasts are generated for entire AD population and for unique subgroups characterized by age groups and the presence of high-impact health conditions (stroke, traumatic brain injury, pneumonia, hypertension, diabetes) prior to AD diagnosis. Then methodology using B-splines in key time points allows is used to analyze scenarios of possible interventions focused on the prevention and treatment of AD/ADRD. Prevalence of AD/ADRD is predicted to be stable between 2017 and 2035 primarily due to a decline in prevalence of pre-AD/ADRD-diagnosis stroke. Mortality, on the other hand, is predicted to increase. In all cases the resulting patterns come from a trade-off of two disadvantageous processes: increased incidence and disimproving survival. The projections are constructed with the assumption that future trends represent a superposition of historic trends in different time periods taken with weights. Sensitivity to assumptions on choice of specific weights are studied, and the approach to choose an optimal combination of weights and therefore, to minimize uncertainties of future forecasts of AD/ADRD prevalence and mortality is suggested and discussed.

